# Nab-paclitaxel plus platinum versus paclitaxel plus platinum as first-line therapy in patients with metastatic or recurrent cervical cancer

**DOI:** 10.1007/s00432-024-05825-z

**Published:** 2024-06-25

**Authors:** Yuan Liu, Shan-shan Fang, Run-sheng Zhao, Bo Liu, Yi-qiang Jin, Quan Li

**Affiliations:** Department of Oncology, Xiangyang Central Hospital, Affiliated Hospital of Hubei University of Arts and Science, No. 136, Jingzhou Street, Xiangyang, Hubei 441021 China

**Keywords:** Metastatic or recurrent cervical cancer, Nab-paclitaxel, Paclitaxel, Chemotherapy

## Abstract

**Purpose:**

This study aimed to assess the efficacy and safety of nanoparticle albumin-bound paclitaxel (nab-paclitaxel) plus platinum versus paclitaxel plus platinum as first-line therapy in patients with metastatic or recurrent cervical cancer.

**Methods:**

Between October 2020 and March 2022, consecutive patients with diagnosed with metastatic or recurrent cervical cancer were retrospectively recruited in our hospital. Fifty-four patients were treated with nab-paclitaxel plus cisplatin or carboplatin. Twenty-four patients were treated with paclitaxel plus cisplatin or carboplatin. A propensity score matching (PSM) analysis was done using a multivariable logistic regression model. The two groups were compared for objective response rate (ORR), progression-free survival (PFS) and overall survival (OS) in the raw and matched dataset.

**Results:**

The nab-paclitaxel group showed a higher ORR than the paclitaxel group both in the raw dataset (72.2% vs. 45.8%; *P* = 0.025) and matched dataset (81.1% vs. 47.6%; *P* = 0.008). The median PFS was significantly longer in the nab-paclitaxel group than in the paclitaxel group both in the raw and matched dataset (12 vs. 7 months; *P* < 0.05). The median OS was not reached in the nab-paclitaxel group compared with 15 months in the paclitaxel group, with a trend toward prolongation. The most common toxicity was hematological adverse events, including grade 3–4 neutropenia, grade 3 anemia and thrombocytopenia in both groups and no statistical differences were observed between the groups (all *P* > 0.05).

**Conclusion:**

Compared with paclitaxel plus platinum, nab-paclitaxel plus platinum may be an effective and tolerable option as first-line therapy for patients with metastatic or recurrent cervical cancer.

## Introduction

Cervical cancer is the fourth most common cancer and the fourth leading cause of cancer-related death among women, with an estimated 604,000 new cases and 342,000 deaths globally in 2020 (Sung et al. 2021). In China, it was estimated that 109,741 new cases of cervical cancer and 59,060 related deaths are estimated to occurred in 2020 (Singh et al. 2023). The incidence rate of cervical cancer is increasing year by year and becoming younger, seriously threatening the health and lives of Chinese women.

Patients with metastatic or recurrent cervical cancer have limited treatment options, and the current standard first-line regimen is paclitaxel combined with cisplatin/carboplatin. However, most patients have a poor prognosis, with a median PFS of < 6 months (Leath and Straughn 2013; Monk et al. 2009). Meanwhile, the traditional solvent-based paclitaxel has limited clinical application due to its tendency to cause allergic reactions and the need for hormonal pretreatment. Therefore, the exploration of new chemotherapy regimens is urgently needed.

Nab-paclitaxel has better pharmacokinetic and pharmacodynamic properties compared to paclitaxel, including faster tumor penetration, lower risk of immunogenicity, and faster recovery from peripheral neuropathy (Desai et al. 2006). Compared to paclitaxel, nab-paclitaxel generally showed significant beneficial effects in terms of ORR, PFS, and OS. Considering its efficacy and tolerability, nab-paclitaxel is gradually replacing paclitaxel as first-line treatment for patients with advanced, recurrent, or metastatic non-small cell lung cancer, breast cancer and pancreatic cancer (Blair and Deeks 2015; Lee et al. 2020; Wang et al. 2022).

Nab-paclitaxel has shown considerable activity and moderate toxicity in the second-line treatment for platinum-resistant, metastatic and recurrent cervical cancer, with the higher efficacy (Alberts et al. 2012; Wang et al. 2023). In recent years, researchers have favored exploring the efficacy of nab-paclitaxel plus platinum in neoadjuvant chemotherapy, concurrent chemoradiotherapy and recurrent or metastatic cervical cancer, which showed promising antitumour activity and a manageable adverse event profile (Jiang et al. 2023; Li et al. 2024; Zhang et al. 2022). Whereas, these are almost single-arm studies, lacking controlled trials compared with traditional paclitaxel.

Therefore, in the present study, we compared the efficacy and safety between nab-paclitaxel plus platinum and paclitaxel plus platinum as firstline therapy in patients with metastatic or recurrent cervical cancer.

## Materials and methods

### Study design and population

The present study was a retrospective observational study. The study protocol was approved by the Research Ethics Committee of Xiangyang Central Hospital (approval number 2021-024). The reporting of the study followed the Strengthening the Reporting of Observational Studies in Epidemiology (STROBE) statement.

Between October 2020 and March 2022, all patients with pathologically diagnosed cervical cancer who received chemotherapy were screened in our hospital. The inclusion criteria included: (1) pathologically diagnosed of cervical cancer; (2) stage IVB or recurrent; (3) age > 18 years; (4) no previous systemic chemotherapy (chemotherapy used as a sensitizer for radiotherapy during radiotherapy was not considered systemic chemotherapy). The exclusion criteria included: (1) presence of other cancers; (2) severe complications out of control; (3) pregnant or lactating women; (4) incomplete clinicopathologic or follow-up data. The clinical data obtained included the age of patients at diagnosis, Eastern Cooperative Oncology Group performance status (ECOG PS), stage of disease, histological type and grade of tumor, treatment history for recurrent patients, presence of comorbidities, chemotherapy regimen administered, profile of treatment toxicity experienced by patients, PFS, OS and the status of the most recent follow-up. The trained attending physicians were responsible for gathering the clinical information pertaining to patients while two senior physicians in the department reviewed criteria for inclusion and exclusion as well as verified patient details and clinical data.

### Treatment

Patients in the nab-paclitaxel group were received nab-paclitaxel 260 mg/m² plus cisplatin 50 mg/m² or carboplatin AUC = 5 on day 1, repeated every 3 weeks. Patients in the paclitaxel group were received paclitaxel 175 mg/m² plus cisplatin 50 mg/m² or carboplatin AUC = 5 on day 1, repeated every 3 weeks. It is important to mention that nab-paclitaxel was often prescribed for patients with a history of food or drug allergies and an underlying hypersensitive constitution. Cisplatin was administered to patients except for those presenting renal insufficiency or compromised cardiac function for whom large volume infusion were unsuitable. Ultimately, the treatment plan was determined based on the patient’s informed consent following a comprehensive understanding. Chemotherapy was given for up to six cycles and was discontinued in cases of progressive disease or unacceptable toxicity, or patient refusal. Dose adjustments were made as follows: (1) neutropenia with fever, grade 4 neutropenia for ≥ 7 days, or grade 4 thrombocytopenia, (2) nephrotoxicity ≥ grade 2, or aspartate aminotransferase (AST), alanine aminotransferase (ALT), alkaline phosphatase (AKP), or bilirubin ≥ grade 3, subsequent treatment was delayed for up to 2 weeks until recovery to grade 1. The dose of chemotherapy drugs in the next cycle was reduced by 20% from the original dose. Patients were discharged if they were still intolerant to chemotherapy with a reduction of 2 dose levels. Individualized radiotherapy during or after chemotherapy was allowed.

### Efficacy and toxicity evaluation

All patients were evaluated for ORR according to Response Evaluation Criteria in Solid Tumors (RECIST) version 1.1. ORR was evaluated every 2 cycles during chemotherapy, which was defined as the percentage of subjects whose overall responses were complete response (CR) or partial response (PR). PFS, OS, and safety were also evaluated. PFS was defined as the time from the start of patient enrollment to tumor progression or death. OS was defined as the time from the start of patient enrollment to death or last follow-up. Safety consisted of all adverse events (AEs), which were assessed for incidence and severity. All AEs were graded according to the National Cancer Institute-Common Toxicity Response Standard (NCI-CTC) version 5.0. All patients were evaluated every 3 months for the first 2 years, and every 6 months for the next 3 years. All patients were followed up until death or October 31, 2023.

### Statistical analysis

Normally distributed continuous variables were presented as means and standard deviations (SDs) and categorical variables were presented as counts and percentages. The comparison of baseline characteristics between the two groups was used t-tests for continuous variables, and a chi-square test or a Fisher exact test for categorical variables. A PSM analysis was done using a multivariable logistic regression model based on: age, stage, pathology, ECOG PS, distant metastasis, prior surgery, prior radiation, prior chemoradiotherapy and combined radiotherapy. Pairs of two groups of patients were derived using 1:2 greedy nearest neighbor matching within caliper of a width equal to 0.05 and performed equilibrium tests. Survival was assessed using Kaplan-Meier curves. The log-rank test was used to compare the survival rates between the two groups. The PSM analysis was performed using STATA 16 (Stata Corp, TX, USA). The experimental data were analyzed by using SPSS 26.0 (IBM Corp, Armonk, NY, USA). GraphPad Prism 9.0 was used for graphing. *P* < 0.05 was defined as being statistically significant.

## Results

### Patient characteristics

Between October 2020 and March 2022, 148 patients with stage IVB or recurrent cervical cancer were admitted to our department. Seventy patients were excluded due to incompatible treatment protocols (*n* = 45), prior systemic chemotherapy (*n* = 17), comorbidities with other malignancies (*n* = 2), treatment interruption or loss to follow-up for which complete clinical data were not available (*n* = 6). Finally, 78 patients were enrolled. There were 54 patients in the nab-paclitaxel group with a median of 6 treatment cycles (range of 3–6 cycles), and 24 patients in the paclitaxel group with a median of 4 treatment cycles (range of 2–6 cycles). The median follow-up time for all patients was 20 months (range of 5–36 months). There were no significant differences in the distribution of the main clinical characteristics between the two groups. And based on the 1:2 matched PSM, there were 37 patients in the nab-paclitaxel group and 21 patients in the paclitaxel group. The overall balance of covariates was better after PSM, with no significant differences between groups (Table 1).

**Table 1:** Characteristics of eligible cervical carcinoma patients in the raw and propensity score-matched dataset

Characteristics	Raw dataset (*n* = 78)			Matched dataset (*n* = 58)		
	Nab-paclitaxelª (*n* = 54)	Paclitaxel^b^ (*n* = 24)	*P*	Nab-paclitaxelª (*n* = 37)	Paclitaxel^b^ (*n* = 21)	*P*
Age, mean ± SD years	51.65 ± 9.968	52.38 ± 7.923	0.753	52.73 ± 9.556	52.81 ± 8.370	0.975
Stage, n (%)			0.298			0.707
IVB	16 (29.6)	10 (41.7)		14 (37.8)	9 (42.9)	
Recurrence	38 (70.4)	14 (58.3)		23 (62.2)	12 (57.1)	
Pathology, n (%)			0.100			0.552
Squamous carcinoma	45 (83.3)	16 (66.7)		29 (78.4)	15 (71.4)	
Non-squamous carcinoma	9 (16.7)	8 (33.3)		8 (21.6)	6 (28.6)	
ECOG PS, n (%)			0.383			0.822
0	17 (31.5)	10 (41.7)		13 (35.1)	8 (38.1)	
1	37 (68.5)	14 (58.3)		24 (64.9)	13 (61.9)	
Sites of disease, n (%)			0.406			0.977
No distant metastasis	26 (48.1)	14 (58.3)		21 (56.8)	12 (57.1)	
Distant metastasis	28 (51.9)	10 (41.7)		16 (43.2)	9 (42.9)	
Prior surgery, n (%)			0.698			0.822
No	34 (63.0)	14 (58.3)		24 (64.9)	13 (61.9)	
Yes	20 (37.0)	10 (41.7)		13 (35.1)	8 (38.1)	
Prior radiotherapy, n (%)			0.583			0.685
No	52 (96.3)	22 (91.7)		35 (94.6)	19 (90.5)	
Yes	2 (3.7)	2 (8.3)		2 (5.4)	2 (9.5)	
Prior chemoradiotherapy, n (%)			0.110			0.544
No	34 (63.0)	20 (83.3)		27 (73.0)	17 (81.0)	
Yes	20 (37.0)	4 (16.7)		10 (27.0)	4 (19.0)	
Combined radiotherapy, n (%)			0.819			0.875
No	24 (44.4)	10 (41.7)		15 (40.5)	8 (38.1)	
Yes	30 (55.6)	14 (58.3)		22 (59.5)	13 (61.9)	

*Notes*^a^Patients received nab-paclitaxel plus cisplatin or carboplatin. ^b^Patients received paclitaxel plus cisplatin or carboplatin

*Abbreviations* SD, standard deviation; ECOG PS, Eastern Cooperative Oncology Group performance status

### Efficacy

Through comprehensive evaluation, the ORR of the nab-paclitaxel group was 72.2% (39/54), 46.3% of patients (25/54) achieved CR, 25.9% of patients (14/54) achieved PR. In the paclitaxel group, the ORR was 45.8% (11/24), 33.3% of patients (8/24) achieved CR, 12.5% of patients (3/24) achieved PR. There was a statistically significant difference in ORR between the two groups (*P* = 0.025). Similarly, the nab-paclitaxel group showed better ORR than the paclitaxel group in the matched dataset (81.1% versus 47.6%, *P* = 0.008) (Table 2).

**Table 2:** Objective response in the raw and propensity score-matched dataset

	Nab-paclitaxelª *n* (%)	Paclitaxel^b^*n* (%)	*P*
Raw dataset (*n* = 78)			
Objective response	39 (72.2)	11 (45.8)	0.025
Matched dataset (*n* = 58)			
Objective response	30 (81.1)	10 (47.6)	0.008

*Notes*^a^Patients received nab-paclitaxel plus cisplatin or carboplatin. ^b^Patients received paclitaxel plus cisplatin or carboplatin

**Fig. 1:**
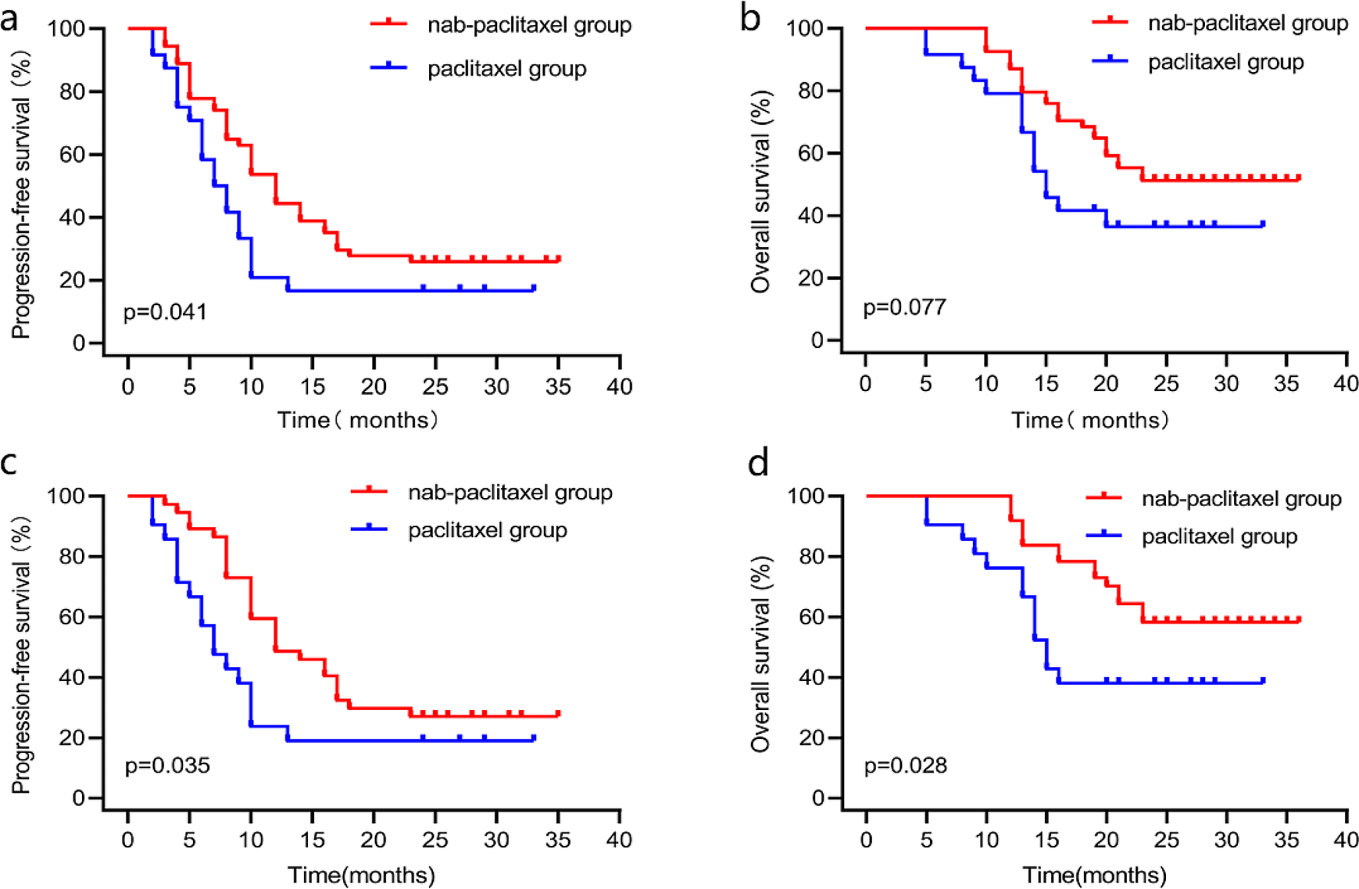
Kaplan–Meier curve of survival in the raw and propensity score-matched dataset. (**a**) Progression-free survival in the raw dataset. (**b**) Overall survival in the raw dataset. (**c**) Progression-free survival in the matched dataset. (**d**) Overall survival in the matched dataset

The median PFS was 12 months (95% CI 9.9–14.1 months) in the nab-paclitaxel group and 7 months (95% CI 4.6–9.4 months) in the paclitaxel group (*P* = 0.041) (Fig. 1a). There was no statistical difference in OS, with the median OS not achieved in the nab-paclitaxel group and 15 months (95% CI 12.6–17.4 months) in the paclitaxel group (*P* = 0.077) (Fig. 1b). Meanwhile, in the matched dataset, the nab-paclitaxel group showed better PFS (12 versus 7 months, *P* = 0.035, Fig. 1c) and OS (not reached versus 15 months, *P* = 0.028, Fig. 1d) than the paclitaxel group.

**Fig. 2:**
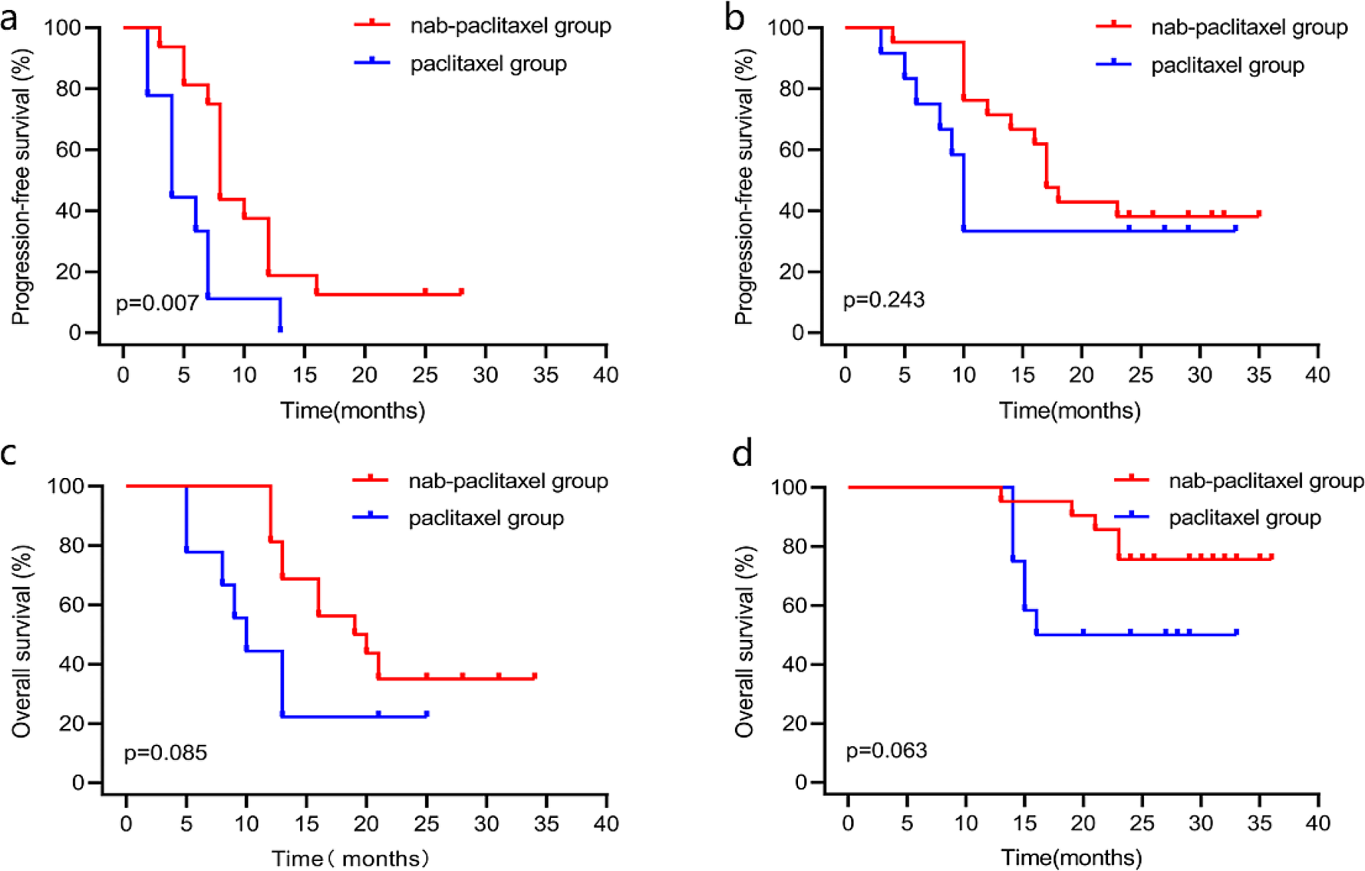
Kaplan–Meier curve of survival for subgroup analysis in the propensity score-matched dataset. (**a**) Progression-free survival for patients with distant organ metastasis. (**b**) Progression-free survival for patients with local recurrence or distant lymph node metastasis. (**c**) Overall survival for patients with distant organ metastasis. (**d**) Overall survival for patients with local recurrence or distant lymph node metastasis

Further subgroup analysis was performed by disease sites in the propensity score-matched dataset. We found that the nab-paclitaxel group had superior PFS to that of the paclitaxel group for patients with distant organ metastasis (8 versus 4 months, *P* = 0.007, Fig. 2a). However, this superiority was not observed in patients with local recurrence or distant lymph node metastasis (17 versus 10 months, *P* = 0.243, Fig. 2b). Although, OS of the nab-paclitaxel group which slightly longer than that of the paclitaxel group for patients in either subgroup was not significantly different (19 versus 10 months, *P* = 0.085, Fig. 2c; not reached versus 18 months, *P* = 0.063, Fig. 2d).

### Toxicity

The most common toxicity was hematological AEs, with grade 3–4 neutropenia occurring in 42.6% of patients (23/54) in the nab-paclitaxel group and 37.5% of patients (9/24) in the paclitaxel group. Meanwhile, the incidences of grade 3 anemia and thrombocytopenia was 13.0% (7/54) and 5.6% (3/54) in the nab-paclitaxel group, and 16.7% (4/24) and 4.2% (1/24) in the paclitaxel group, respectively, and no grade 4 anemia and thrombocytopenia were occurred. No significant differences were observed between the two groups (all *P* > 0.05). Non-hematological toxicities were generally not severe and most were manageable. The incidence of grade 1–2 peripheral neurotoxicity was 57.4% (32/54) in the nab-paclitaxel group and 45.8% (10/24) in the paclitaxel group (*P* > 0.05). In the paclitaxel group, two patients had mild allergic reactions due to paclitaxel, whereas no cases of allergy were observed in the nab-paclitaxel group. Besides, dose reductions occurred in 16 patients receiving nab-paclitaxel (29.6%) and eight patients receiving paclitaxel (33.3%). There were no treatment-related deaths in either group.

## Discussion

This study was a real-world retrospective study, the first to systematically evaluate the efficacy and safety of nab-paclitaxel plus platinum versus paclitaxel plus platinum as firstline therapy for patients with metastatic or recurrent cervical cancer. We observed that ORR and PFS of nab-paclitaxel plus platinum was significantly better than those of paclitaxel plus platinum both in the raw and propensity score-matched dataset. Subgroup analyses showed a significant improvement in PFS with nab-paclitaxel plus platinum over paclitaxel plus platinum in patients with distant organ metastasis. In regard to AEs, the two regimens were comparable.

Based on our current knowledge, nab-paclitaxel plus platinum demonstrated the highest numerical response rate and longest PFS evaluating first-line chemotherapy for metastatic or recurrent cervical cancer. Specifically, it achieved a noteworthy ORR of 72.2% and a median PFS of 12 months, indicating significant progress compared to using paclitaxel plus platinum. Subgroup analyses further indicated the value of first-line nab-paclitaxel plus platinum for patients with distant organ metastasis. In order to minimize potential interference arising from the baseline characteristics of both groups (despite their relatively balanced initial distribution), we additionally employed the PSM technique. The observed disparities in ORR and PFS between the two groups remained statistically significant, thus ensuring the reliability of our findings. Despite the observed trend of prolonged median OS in the nab-paclitaxel group, there was inconsistency in the difference of median OS between the two groups in the raw and propensity score-matched dataset. The potential factors contributing to this interference in results may be attributed to inadequate sample size and insufficient duration of follow-up. Therefore, it is still unclear whether the improvement in ORR and PFS can translate into substantial OS benefits. Preclinical study suggested that nab-paclitaxel demonstrated enhanced antitumor activity and higher intracellular concentrations compared to conventional paclitaxel when administered at the same dosage (Desai et al. 2006). Some studies have also shown that cervical cancer cells exhibited significant expression of secreted protein acidic and rich in cysteine (SPARC) in both the cytoplasm and extracellular matrix. Furthermore, high SPARC expression was positively correlated with factors such as poor differentiation, advanced stages, and lymph node metastasis in cervical cancer, and served as an unfavorable prognostic marker (Chen et al. 2012; Han et al. 2023; Shi et al. 2016). The strong affinity between SPARC and albumin contributed to the enhanced utilization of nab-paclitaxel by tumor cells, thereby potentially augmenting the therapeutic efficacy (Pascual-Pasto et al. 2022; Tai and Tang 2008). In addition, the toxicity of nab-paclitaxel was reduced compared to conventional paclitaxel, enabling an increase in the administered dose by 50% (Gradishar et al. 2005; Ibrahim et al. 2002). These factors may account for the observed increase in antitumor efficacy of nab-paclitaxel compared to paclitaxel.

In the study by Nabholtz et al., paclitaxel at a dose of 175 mg/m² revealed superior efficacy over 135 mg/m², suggesting a dose-response relationship (Nabholtz et al. 1996), and was then widely accepted as the dosage standard. Our study using 175 mg/m² paclitaxel as control showed an ORR of 45.8%, a median PFS of 7 months (95% CI 4.6–9.4 months) and a median OS of 15 months (95% CI 12.6–17.4 months), similar to the conclusions drawn by Takekuma et al. (Takekuma et al. 2012). A phase II trial of 175 mg/m² nab-paclitaxel plus nedaplatin for advanced, recurrent, or metastatic cervical cancer indicated an ORR of 50.0%, a median PFS of 9.1 months (95% CI 2.4–15.8 months) and a median OS of 16.6 months (95% CI 12.6–20.6 months) (Li et al. 2017). Another study showed an ORR of 40% and a duration of response (DOR) of 6.7 months with camrelizumab plus nab-paclitaxel and carboplatin (Zhang et al. 2022). In the current study, we found the patients in nab-paclitaxel plus platinum had better ORR and PFS compared to data from previous studies, and the median OS was not achieved. These results may be explained by three main factors: (i) the nab-paclitaxel dose intensity in this study was 260 mg/m²; (ii) this study did not include patients who had received previous systemic chemotherapy and therefore did not include platinum-resistant patients, who had a very poor prognosis (Vergote et al. 2023); (iii) individualized radiotherapy during or after chemotherapy was allowed, thus reflecting the partly combined effect of chemotherapy and radiotherapy, not the effect of chemotherapy alone. The findings were comparable to other previously reported studies, which suggested that chemotherapy plus salvage radiotherapy was an effective treatment for recurrent or metastatic cervical cancer, especially for distant lymph node metastasis or oligometastases to three or fewer sites (Chopra et al. 2022; Kim et al. 2018). However, we did not observe such a difference in the paclitaxel group, and radiotherapy did not differ at baseline between the two groups, inferring that nab-paclitaxel combined with radiotherapy was more likely to exert an efficacy advantage, similar to the previous report (Yu et al. 2021).

Concerning grade ≥ 3 AEs, the most common toxicity was hematological AEs, consistent with that in previous reports (Alberts et al. 2012; Li et al. 2017). Although, in the GOG 204 study, grade 3–4 neutropenia occurred in 78.2% of patients (Monk et al. 2009). The difference possibly because of prophylactic use of polyethylene glycol recombinant human granulocyte colony-stimulating factor. We also found that grade 1–2 peripheral neurotoxicity was common. With a higher rate of neurotoxicity in the nab-paclitaxel group, this difference was not statistically significant. A previous meta-analysis also indicated that the incidence of neurotoxicity induced by nab-paclitaxel compared to paclitaxel was trending higher in the high-dose arm (≥ 150mg/m^2^), but significantly higher in the low-dose arm (< 150mg/m^2^) (Guo et al. 2019). In addition, despite the administration of corticosteroid and antihistamine premedication, the incidence of allergic reactions to paclitaxel exceeded that observed with nab-paclitaxel with no premedication. Patients with a history of food or drug allergies and heightened sensitivity tended to favor nab-paclitaxel when considering treatment options, even potentially reducing the discrepancy in allergic reaction incidence between the two groups. Overall, the toxicities were almost comparable between the two groups.

The present study is subject to certain limitations. First, this retrospective study was conducted at a single institution, and caution should be exercised when extrapolating the results to other cervical cancer patients due to potential institutional bias in patient demographics. Second, the non-random patient selection for treatment option gave rise to potential selection bias that cannot be disregarded. Although we employed PSM statistical methods to account for baseline characteristics between the two groups, some unobserved confounding factors might still exist and could potentially introduce bias into our study findings. Third, the robustness of the results, particularly in subgroup analysis and evaluation of long-term survival, might be compromised due to the limited sample size and insufficient duration of follow-up.

## Conclusion

Compared with paclitaxel plus platinum, nab-paclitaxel plus platinum had a significantly higher ORR and PFS with manageable toxicity as firstline therapy for patients with metastatic or recurrent cervical cancer. As a retrospective study with limited sample size and follow-up, the study found a trend toward prolonged OS with this regimen. Prospective, large-sample, multicenter randomized controlled trials are needed to further confirm our results. And the regimen may also be the appropriate chemotherapy backbone to explore the addition of novel agents.

## Data Availability

No datasets were generated or analysed during the current study.
